# Tear film break-up patterns in thyroid eye disease

**DOI:** 10.1038/s41598-021-84661-4

**Published:** 2021-03-05

**Authors:** Yasuhiro Takahashi, Patricia Ann L. Lee, Aric Vaidya, Shinjiro Kono, Hirohiko Kakizaki

**Affiliations:** 1 Department of Oculoplastic, Orbital and Lacrimal Surgery, Aichi Medical University Hospital, 1-1 Yazako-Karimata, Nagakute, Aichi 480-1195 Japan; 2 Rapti Eye Hospital, Dang, Nepal

**Keywords:** Corneal diseases, Thyroid diseases

## Abstract

Evaluation of tear film break-up pattern (TFBUP) is the main diagnostic method for tear film-oriented therapy (TFOT) of dry eye. This prospective, observational study examined TFBUPs in 154 eyes/sides from 78 patients with thyroid eye disease (TED) who met the diagnostic criteria for dry eye in Japan. TFBUPs were classified as area, line, spot, dimple, and random breaks. Results for the status of TED and dry eye were compared between the TFBUPs. Consequently, line, spot, dimple, and random breaks were observed in 80 (51.9%), 29 (18.8%), 10 (6.5%), and 35 eyes (22.7%) while no eyes showed area breaks. The random break group had the highest incidence of lid lag/Graefe sign and superior limbic keratoconjunctivitis (SLK) (P < 0.050). Although the incidence of each TFBUP is almost equal in patients with simple dry eye without TED, line breaks were more frequently observed in TED. In addition, while random breaks in simple dry eye are usually only associated with minor ocular surface damages, those in TED were associated with a higher incidence of concomitant SLK. These results will be helpful for understanding the etiology of dry eye in TED and for TFOT in TED.

## Introduction

Tear film-oriented diagnosis (TFOD) and therapy (TFOT) are new concepts in the diagnosis and treatment of dry eye^1^. The tear film consists of lipid and mucoaqueous-gel layers including aqueous tears and secretory mucins^1^. Its stability is maintained by the ocular surface epithelium and by membrane-associated mucins in the glycocalyx layer^1^. When any of these components becomes dysfunctional, the vicious cycle of the dry eye begins^1^. TFOD enables ophthalmologists to identify not only the dysfunctional component, but also differentiate between the aqueous-deficient dry eye, decreased-wettability dry eye, and increased-evaporation dry eye^1^. This provides an appropriate treatment of dry eye through TFOT^1^.

Although the evaluation of tear film break-up pattern (TFBUP) is simple and non-invasive, it is the main diagnostic method for TFOD^1,2^. TFBUPs are classified by tear film dynamics into five patterns: area, line, spot, dimple, and random breaks^1,2^. The area and line breaks indicate aqueous-deficient dry eye. Both are characterized by short tear film break-up time (TFBUT) and low tear meniscus height (TMH), but the area breaks are seen in the more severe aqueous-deficient dry eye^1,2^. The spot and dimple breaks imply decreased tear film stability and corneal surface wettability. However, aqueous tear volume and tear film lipid layer properties remain normal in dimple breaks^2^. Random breaks are the mildest manifestation of dry eye and indicate increased tear evaporation^1,2^. The incidence of each pattern is almost the same in patients with a simple dry eye^2^.

The patients with a thyroid eye disease (TED) have a high prevalence of dry eye; 65–85% of them complain of dry eye symptoms^3^. Several characteristic mechanisms are associated with the development of dry eye in TED. These include reduced aqueous tear production due to lacrimal gland involvement; excess tear evaporation due to increased ocular surface exposure; meibomian gland dysfunction due to incomplete blinking; and abnormal friction between the ocular surface and eyelid due to increased eyelid pressure^3–6^. These may produce different clinical features in terms of TFBUPs and change the incidence of each TFBUP as seen in simple dry eye without TED.

In this study, we examined the TFBUPs in TED and compared them to those in dry eye without TED.

## Results

All data are included in the supplementary file. Patient data are shown in Table 1. This study included 154 eyes/sides from 78 patients (26 men and 52 women; mean age, 41.2 ± 13.4 years; range, 19–74 years). Twenty eyes/sides of ten patients were excluded from this study because of use of contact lenses, history of orbital decompression surgery, or history of orbital radiotherapy. We also excluded four eyes/sides from three patients who did not meet the diagnostic criteria for dry eye in Japan^1,7^. All patients were previously diagnosed with thyroid dysfunction before referral by endocrinologists at other clinics, and 74 patients received oral treatment, including thiamazole, propylthiouracil (PTU), potassium iodide, and/or levothyroxine. Twenty-five and six patients continued to exhibit signs of hyperthyroidism and hypothyroidism, respectively, while the remaining 47 patients were considered as controlled euthyroid patients. At least one of the thyroid autoantibodies, including thyroid stimulating hormone receptor antibody (TRAb), thyroid stimulating antibody (TSAb), anti-thyroid peroxidase antibody (TPOAb), and/or anti-thyroglobulin antibody (TgAb), was positive in 75 patients, while the other three patients had shown negative conversion of thyroid autoantibodies. Fifteen patients were smokers at the time of examination or stopped smoking less than 2 years before the examination. Nine patients had a history of steroid treatment for active TED.

**Table 1:** Patient data.

	Total
Number of patients/sides	78/154
Age (years)	41.2 ± 13.4
**Sex (M/F)**	
M	26 (33.3%)
F	52 (66.7%)
**Thyroid status (patients)**	
Hyperthyroid	25 (32.1%)
Euthyroid	47 (60.3%)
Hypothyroid	6 (7.7%)
**Smoking (patients)**	
0 (non-smoker)	63 (80.8%)
1 (< 10 cigarettes/day)	5 (6.4%)
2 (10–20 cigarettes/day)	7 (9.0%)
3 (> 20 cigarettes/day)	3 (3.8%)
CAS	0.6 ± 0.8
**Lid lag/Graefe sign (sides)**	
Presence	59 (38.3%)
Absence	95 (61.7%)
MRD-1 (mm)	3.8 ± 1.3
MRD-2 (mm)	5.8 ± 0.8
Hertel exophthalmometric value (mm)	16.0 ± 3.2
TFBUT (s)	1.6 ± 0.9
**AD classification**	
A	0.8 ± 0.9
D	1.0 ± 1.1
**SLK (eyes)**	
Presence	57 (37.0%)
Absence	97 (63.0%)
Schirmer's test (mm)	16.1 ± 10.5
**TMH (μm)**	
Upper	269.0 ± 79.8
Lower	316.3 ± 182.0
DEQS	24.5 ± 21.1

*M* male, *F* female, *CAS* clinical activity score, *MRD* margin reflex distance, *TFBUT* tear film break-up time, *SLK* superior limbic keratoconjunctivitis, *TMH* tear meniscus height, *DEQS* dry eye-related quality-of-life score.

**Figure 1:**
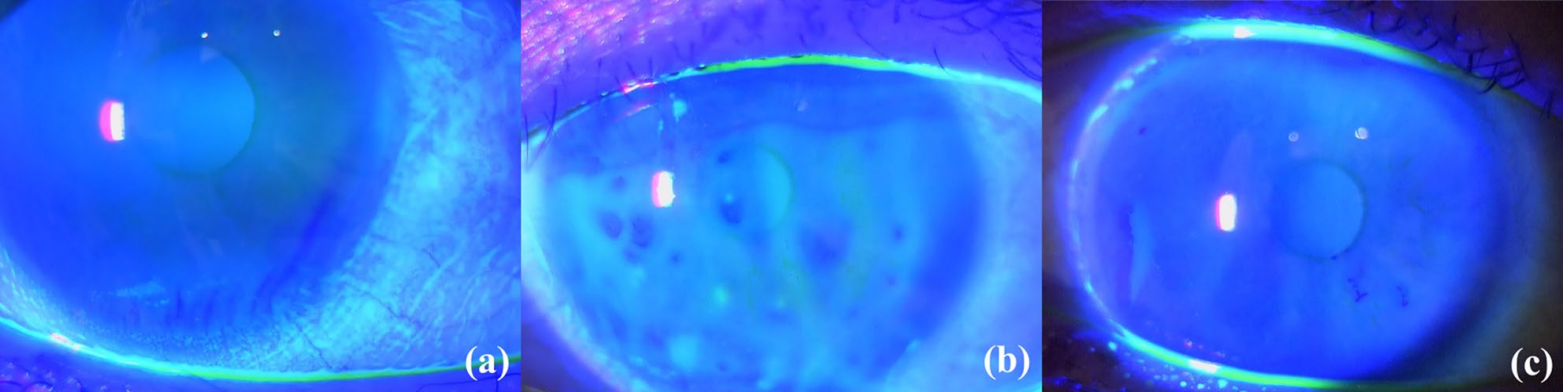
Tear film break-up patterns in thyroid eye disease. (**a**) Line break, (**b**) spot break, (**c)** dimple break.

**Figure 2:**
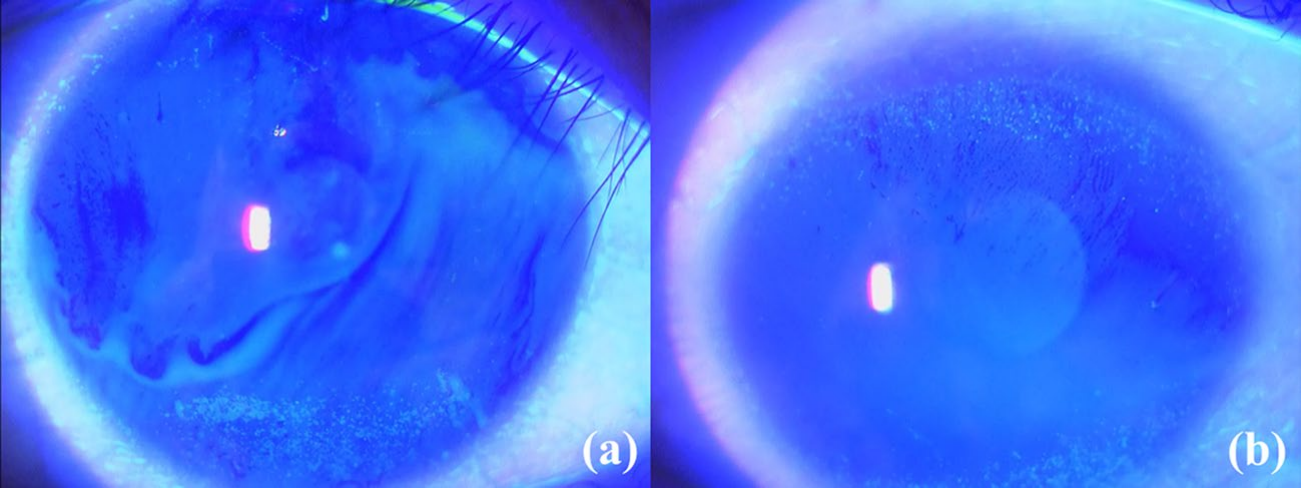
Random breaks in thyroid eye disease. Tear film break-up occurs simultaneously in the upper and lower parts of cornea (**a**) or occurs from the upper part of cornea (**b**).

The results of measurements and statistical comparisons are shown in Tables 1 and 2. The TFBUPs such as line, spot, dimple, and random breaks were demonstrated in 80 (51.9%), 29 (18.8%), 10 (6.5%), and 35 eyes (22.7%), respectively (Figs. 1 and 2). None of the eyes showed the area break pattern. The distribution of the TFBUPs was significantly different between the present study and the previous study reported by Yokoi et al.^2^ (P < 0.001). Sixteen sides/eyes showed clinical activity score (CAS) ≥ 3 points or extraocular muscle inflammation on magnetic resonance images, and the distribution was not different between eyes/sides in the active and inactive phases of TED (P = 0.320). Thirty-seven of 76 patients (48.7%) who were examined in both eyes/sides showed different TFBUPs between the eyes.

**Table 2:** Comparison of the results among the tear film break-up pattern (TFBUP) groups.

TFBUP	Line	Spot	Dimple	Random	P value
Number of eyes	80 (51.9%)	29 (18.8%)	10 (6.5%)	35 (22.7%)	
CAS	0.6 ± 0.8	0.8 ± 0.9	0.4 ± 0.7	0.3 ± 0.7	0.080
**Lid lag/Graefe sign (sides)**					
Presence	22 (27.5%)	15 (51.7%)	2 (20.0%)	20 (57.1%)	0.005
Absence	58 (72.5%)	14 (48.3%)	8 (80.0%)	15 (42.9%)	
MRD-1 (mm)	3.8 ± 1.2	3.8 ± 1.6	4.2 ± 1.1	3.8 ± 1.2	0.782
MRD-2 (mm)	5.8 ± 0.8	6.0 ± 0.9	5.9 ± 0.4	5.6 ± 0.7	0.225
Hertel exophthalmometric value (mm)	15.7 ± 3.2	16.7 ± 3.3	15.7 ± 3.4	16.3 ± 3.2	0.523
TFBUT (s)	1.6 ± 0.9	1.2 ± 0.7	2.2 ± 1.1	1.7 ± 1.0	0.013
**AD classification**					
A	0.7 ± 0.8	0.7 ± 0.8	0.4 ± 0.5	1.2 ± 1.1	0.048
D	0.9 ± 1.1	0.8 ± 1.0	0.9 ± 1.3	1.3 ± 1.2	0.305
**SLK (eyes)**					
Presence	22 (27.5%)	13 (44.8%)	2 (20.0%)	20 (57.1%)	0.011
Absence	58 (72.5%)	16 (55.2%)	8 (80.0%)	15 (42.9%)	
Schirmer's test (mm)	16.4 ± 10.2	16.3 ± 11.4	17.1 ± 12.7	15.3 ± 10.2	0.947
**TMH (μm)**					
Upper	268.7 ± 85.3	285.3 ± 75.0	252.2 ± 61.8	261.0 ± 75.6	0.573
Lower	320.5 ± 165.6	380.6 ± 286.5	267.2 ± 59.6	267.5 ± 100.0	0.073

*CAS* clinical activity score, *MRD* margin reflex distance, *TFBUT* tear film break-up time, *SLK* superior limbic keratoconjunctivitis, *TMH* tear meniscus height.

Regarding the TED-associated findings, the CAS tended to be higher in the spot break group than the random break group, although the difference did not reach statistical significance (P = 0.062). The lid lag/Graefe sign was more frequently observed in the spot and random break groups (P = 0.005). The margin reflex distance (MRD)-1 and -2, and Hertel exophthalmometry values were not significantly different between the groups (P > 0.050).

As for the dry eye findings, TFBUT was longer in the dimple break group than in the spot break group (P = 0.013). The D classification score, the results of Schirmer’s test, and the upper TMH were not significantly different between the groups (P > 0.050). However, the A classification score was significantly higher in the random break group (P = 0.048), and 20 of the 35 eyes (57.1%) in the random break group showed superior limbic keratoconjunctivitis (SLK) (P = 0.011). Seventeen of these 20 patients showed simultaneous tear break-up in both the upper and lower parts of the cornea or that starting from the upper part of the cornea (Fig. 2). The lower TMH was lesser in the random break group than the spot break group, although the difference did not reach statistical significance (P = 0.063).

Similarly, mild, moderate-to-severe, and sight-threatening TED were seen in 59 (38.3%), 93 (60.4%), and 2 (1.3%) eyes/sides, respectively (Table 3). The ratio of TFBUP in each TED severity group was not different (P = 0.287).

**Table 3:** Ratio of tear film break-up patterns among severity of thyroid eye disease.

Number of eyes	Line	Spot	Dimple	Random
Mild	35	9	1	14
Moderate-to-severe	43	20	9	21
Sight-threatening	2	0	0	0

## Discussion

This study examined the TFBUPs in TED and compared these patterns to those associated with dry eye. Line break was the most frequently observed pattern, followed by random, spot, and dimple breaks. None of the eyes showed area break, which is the most severe manifestation of aqueous-deficient dry eye^1,2^. Although a previous study showed that the incidence of each pattern is almost equal in patients with a simple dry eye without TED^2^, the distribution of TFBUPs was significantly different between the results in the present and previous studies (P < 0.001)^2^. This indicates that TED more frequently induces aqueous-deficient dry eye, probably due to lacrimal gland involvement; however, it is found to be relatively mild.

In the simple dry eye without TED, the random break is the mildest manifestation and allude to minor ocular surface damages due to increased tear evaporation^1,2^. However, the random break group in this study showed a higher A classification score (P = 0.048) and a higher incidence of SLK (P = 0.011). In contrast, since MRD-1 and -2, and Hertel exophthalmometry values were not significantly different among the TFBUP groups (P > 0.050), ocular surface exposure and tear evaporation in the random break group were probably similar to those in the other groups. Also, in 17 of the 20 patients with SLK, tear break-up occurred from the upper part of the cornea or simultaneously in both the upper and lower parts of the cornea (Fig. 2). These random breaks may be other TFBUPs characterized by TED. A previous study demonstrated that upper eyelid retraction and high MRD-1 were indicators for resistance to treatment for SLK because these cause high upper eyelid pressures on the ocular surface and result in an abnormal friction between the ocular surface and the eyelid^6^. As the etiology of lid lag and Graefe sign, which were more frequently demonstrated in the random break group (P = 0.005), is similar to that of upper eyelid retraction, high upper eyelid pressures may cause tear film break-up predominantly in the upper cornea, resulting in those unique random breaks.

The results of Schirmer’s test I and TMH were not significantly different (P > 0.050) among the line, spot, and dimple break groups, but TFBUT was longer in the dimple break group than the other groups (P = 0.013). These findings were similar to those seen in the simple dry eye^2^. In contrast, the D classification score showed no significant difference between these three groups. This was different from the results published in a previous study, which showed that the line break group was associated with a higher conjunctivo-corneal staining scores in the simple dry eye^2^. Based on the low D classification score in the line break group in TED, the line break may indicate a relatively mild aqueous-deficient dry eye, as mentioned above.

The spot break group showed a tendency towards a higher CAS than the random break group (P = 0.062). Spot breaks indicate low wettability of the ocular surface due to deficiency of membrane-associated mucins. The mucins are secreted by goblet cells^8^, which may be impaired due to ocular surface inflammation in TED^9^. This may be expressed as a higher CAS in the spot break group.

In general, Japanese patients present a low CAS, even if they have inflamed extraocular muscles confirmed on magnetic resonance images^10^. This may reflect an averagely low CAS in all the groups.

Patients with moderate-to-severe TED have wider palpebral fissure heights, more proptotic eyes, and a higher incidence of lagophthalmos, resulting in more tear evaporation. However, in this study, the severity of TED was not associated with the TFBUPs.

Several options have been reported for the treatment of dry eye in TED^3^. Based on the results of our study, line breaks were most frequently seen. The eyes with line breaks can benefit from artificial tears and lubricant eyedrops, which increase aqueous volume^3^. Diquafosol sodium promotes secretion of both aqueous tears and secretory mucins^2,11^ and may be the best option for this dry eye pattern. For patients with low wettability resulting in spot and dimple break patterns, topical diquafosol and rebamipide can provide adequate treatment since both the medications increase the amounts of membrane-associated mucins^2,6^. Rebamipide also has an anti-inflammatory effect^6^, which may be beneficial for patients with spot breaks and high CAS. Since random breaks in TED do not appear to be associated with excess tear evaporation, as mentioned previously, topical rebamipide may be a better option to treat SLK in this group^6^.

Our study was limited by several factors. This study unexpectedly included only a small number of patients in the sight-threatening group, those in the active phase of TED, those with eyelid retraction, and those with severe proptosis. It did not include patients with a simple dry eye without TED as controls. Although we compared the distribution of the TFBUPs, analysis of the influence of TED on TFBUPs using raw data with logistic regression analysis would provide a more precise information. We could not collect precise data on thyroid autoantibody levels. As a recent study showed the correlation between TRAb level and CAS^12^, TRAb level may have an influence on TFBUPs. Also, we did not evaluate the tear film lipid layer components, which may provide more information on dry eye. Similarly, we did not examine the ocular surface area and blink rate, data of which may provide an additional information on tear evaporation. Furthermore, all examinations were performed by a single examiner, which may cause an examiner bias in this study.

In conclusion, TFBUPs in patients with TED appeared to be different from those observed in the simple dry eye without TED. The results in this study will help understand the etiology of dry eye in TED and for TFOT in TED.

## Materials and methods

### Study design and ethics approval

This prospective, observational study was approved by the institutional review board (IRB) of Aichi Medical University Hospital (No. 15-043 and 2020-087). This study was also conducted in accordance with the tenets of the Declaration of Helsinki and its later amendments. The IRB granted a waiver of informed consent for this study based on the ethical guidelines for medical and health research involving human subjects established by the Japanese Ministry of Education, Culture, Sports, Science, and Technology; and by the Ministry of Health, Labour, and Welfare. The waiver was granted because the study was not an interventional study. Nevertheless, at the request of the IRB, we published an outline of the study on the Aichi Medical University website to give patients the opportunity to refuse to participate in the study, although none did. Personal identifiers were removed from the records prior to data analysis.

### Patients

This study included consecutive Japanese patients with TED seen by one of the authors (YT) between January 2015 and October 2015 at Aichi Medical University Hospital. A diagnosis of TED was made based on the presence of at least one characteristic sign (eyelid fullness, eyelid retraction, proptosis, and/or restrictive strabismus) and the presence of thyroid autoimmunity^13–15^. We also included eyelid lag and Graefe sign as the characteristic signs because these are associated with eyelid retraction^16^, but some patients with lid lag/Graefe sign do not show eyelid retraction. Both eyes/sides were included in this study because TED can present differently between the two eyes/sides in the same patient^17^. We excluded patients who wore contact lenses, used topical eyedrops for dry eye and/or glaucoma, had a history of intraocular, eyelid, or orbital surgery, had undergone orbital radiotherapy, and those with systemic diseases affecting the ocular surface except thyroid dysfunction^18^. Although there is a high prevalence of meibomian gland dysfunction in TED patients^4^, we excluded patients with severe meibomian gland dysfunction characterized by at least 1 of the following: the presence of an irregular eyelid margin, toothpaste-like meibum expression with more than moderate eyelid compression or no expression even with hard compression, a Marx line score ≥ 7 points, or loss of meibomian gland area ≥ two-thirds visualized through a meibography^19^ to match patients’ condition with that in a previous study^2^. We also excluded patients who did not meet the diagnostic criteria for dry eye syndrome in Japan, including TFBUT ≤ 5 s and presence of dry eye-associated symptoms^1,7^.

### Data collection

The following data were collected: patient age, sex, thyroid function, presence or absence of thyroid autoantibodies, treatment for TED, the period from the onset of TED to the time of examination, and smoking status. Smoking status was classified as the number of cigarettes smoked per day as follows: 0, no smoking; 1, ˂ 10 cigarettes/day; 2, 10–20 cigarettes/day; and 3, ˃ 20 cigarettes/day^20^. Patients who previously smoked but stopped smoking cigarettes ≥ 2 years prior to examinations were considered as non-smokers^21^ (Supplementary information).

### Evaluation of TED-associated findings

All the evaluations and examinations were performed by one of the authors (YT). The following items were evaluated and measured: severity of TED, CAS, the presence or absence of lid lag and/or Graefe sign, MRD-1 and -2, and Hertel exophthalmometry value.

### Severity of TED

The severity of TED is usually classified according to the EUGOGO consensus statement^22^; however, Japanese patients generally present with milder symptoms^23^. Classifying severity in this study was, therefore, based on the proposal by the Japanese Committee for Diagnostic Criteria and Guideline to Medical Care for Thyroid-Associated Malignant Exophthalmos, as being: mild, if with milder manifestations than moderate-to-severe cases; moderate-to-severe, if with palpebral fissure height ≥ 10 mm, moderate-to-severe soft tissue involvement, lagophthalmos, proptosis ≥ 18 mm, and/or diplopia in any gaze; or sight-threatening, if with corneal ulcer or perforation, and/or compressive optic neuropathy^10^. Compressive optic neuropathy was diagnosed based on optic nerve compression seen on imaging in the absence of other causes of vision loss and the presence of at least one of the following: visual acuity of 0.5 or less, positive relative afferent pupillary defect, central or paracentral scotoma, and papilloedema or a pale optic disc^24^.

### CAS

CAS was calculated using seven parameters: retrobulbar discomfort, pain on eye movement, eyelid erythema, eyelid swelling, conjunctival injection, chemosis, and caruncle swelling^25^.

### TED activity

We judged eyes/sides in the active phase of TED when CAS was three points or more^25^ or the extraocular muscles were inflamed as confirmed on magnetic resonance images.

### Lid lag/Graefe sign

The presence or absence of lid lag and/or Graefe sign was confirmed in each eyelid. Lid lag is a static condition in which the affected eyelid is higher than normal while the eye is in downgaze^16^. Graefe sign is a dynamic phenomenon wherein the affected eyelid lags behind on downward rotation of the eye^16^.

### MRD

MRD-1 and -2 were measured as the distance from the upper (MRD-1) or lower eyelid margin (MRD-2) to corneal light reflex in the primary gaze. While seated and with the brow fixed, the patient was requested to look at a light source (a pen torch); distances were then recorded using a millimeter ruler^26^.

### Hertel exophthalmometry

Hertel exophthalmometry measures the distance from the corneal apex to a plane defined by the deepest point on the lateral orbital rim.

### Evaluation of dry eye

The following items were evaluated and measured: TFBUP, TFBUT, AD classification scores of fluorescein corneal staining, presence or absence of SLK, Schirmer’s test I, TMH, and dry eye-related quality-of-life score (DEQS).

### TFBUPs

TFBUP was classified into the following five patterns: area, line, spot, dimple, and random breaks^2,27^. The area break was diagnosed when the upward movement of fluorescein was not observed or was limitedly observed in the lower cornea upon eyelid opening^2^. The line break was diagnosed when a vertical line-shaped area of fluorescein was formed in the lower cornea immediately upon eyelid opening and in which intensity decreased as upward movement of fluorescein was completed^2^. The spot break was diagnosed when the spot-shaped areas of fluorescein formed after eyelid opening, where at least one spot remained through the upward movement of dye^2^. Similarly, dimple break was diagnosed when an irregular but vertical line-shaped area of fluorescein formed near the central cornea and in which intensity increased until the upward movement of fluorescein was completed^2^. The random break was diagnosed when the fluorescein pattern formed could not be classified into the other four types^2^. It generally occurs after the cessation of fluorescein upward movement^2^.

At the time of fluorescein staining, strict attention was paid not to increase the subjects’ tear volumes^2^. To determine TFBUP, a drop of physiological saline solution was put on a fluorescein test strip. After an excess saline solution was shaken off the strip, the strip was gently touched to the center of the lower eyelid margin. The TFBUP was confirmed thrice in each patient.

### TFBUT

TFBUT was measured similarly to that of the evaluation of TFBUP. The time from the eyelid opening to the first appearance of a dry spot on the tear film was measured.

### AD classification scores

The area (A) and density (D) classification of corneal fluorescein staining was graded using the scale reported by Miyata et al.^28^. The A was classified as follows: grade 0, no punctate staining; grade 1, the staining involving less than one-third of the cornea; grade 2, the staining involving one-third to two-thirds of the cornea; and grade 3, the staining involving more than two-thirds of the cornea^28^. The D was classified as follows: grade 0, no punctate staining; grade 1, sparse density; grade 2, moderate density; and grade 3, high density and overlapped lesion^28^.

### SLK

Patients with at least two of the following criteria were diagnosed with SLK: blood vessel dilation in the superior bulbar conjunctiva, papillary inflammation of the upper tarsal conjunctiva, punctate fluorescein staining of the superior conjunctiva and the upper cornea, filaments in the upper cornea, epithelial thickening of the superior bulbar conjunctiva, and redundancy of the superior bulbar conjunctiva^6^.

### Schirmer’s test I

The Schirmer’s test I was performed without topical anaesthesia as follows: a Schirmer’s test strip was placed on the lower conjunctival sac without touching the cornea. The length of the wet portion was measured after 5 min.

### TMH

TMH was measured on the sagittal plane through the center of the upper and lower eyelids using optical coherence tomography (RS-3000, NIDEK CO., LTD, Aichi, Japan)^29,30^. The dedicated attachment was used to observe the anterior segment of the eyes.

### DEQS

All patients filled in the 15-item DEQS questionnaire^31,32^. It consists of two subscales, the “bothersome effects of ocular symptoms (6 items)” and the “impact of dry eye on daily life (9 items)” scores. The DEQS asks patients for the frequency (A-column) and severity of each item (B-column). We calculated the DEQS score as follows: the sum of scores in the B-column × number of valid responses × 25. All the scores ranged from 0 to 100, with a higher score representing a greater disability.

### Statistical analysis

Patients’ age and measurement values were expressed as the mean value ± standard deviation. The eyes were classified into different groups, based on the TFBUP. The CAS, MRD-1 and -2, Hertel exophthalmometry value, TFBUT, Schirmer’s test I results, and TMH were compared between the patient groups using one-way ANOVA and Tukey–Kramer post hoc test. The AD classification scores were compared between the patient groups using the Kruskal–Wallis test. The distribution of TFBUPs was compared between the present study and the previous study reported by Yokoi et al.^2^ using a chi-square test. That was also compared between the eyes/sides in the active and inactive phases of TED using a chi-square test. The ratios of presence or absence of lid lag/Graefe sign and SLK were compared between the patient groups using a chi-square test. All statistical analyses were performed using SPSS version 26 software (IBM Japan, Tokyo, Japan). A P-value of < 0.05 was considered statistically significant.

## Supplementary Information

Supplementary Table.

## Data Availability

We submit a supplemental file including all data.
